# Supplementary material for: Causal Relationship between Aging and Anorexia Nervosa: A White-Matter-Microstructure-Mediated Mendelian Randomization Analysis
Source: Biomedicines. 2024 Aug 16;12(8):1874. doi: 10.3390/biomedicines12081874 (PMC11351342; doi:10.3390/biomedicines12081874)
Supplement: Supplementary file 1 [file biomedicines-12-01874-s001.zip › Supplementary Figure/Figure S4/Singe SNP plot of MR analysis for TL to ubm-b-1538.pdf]

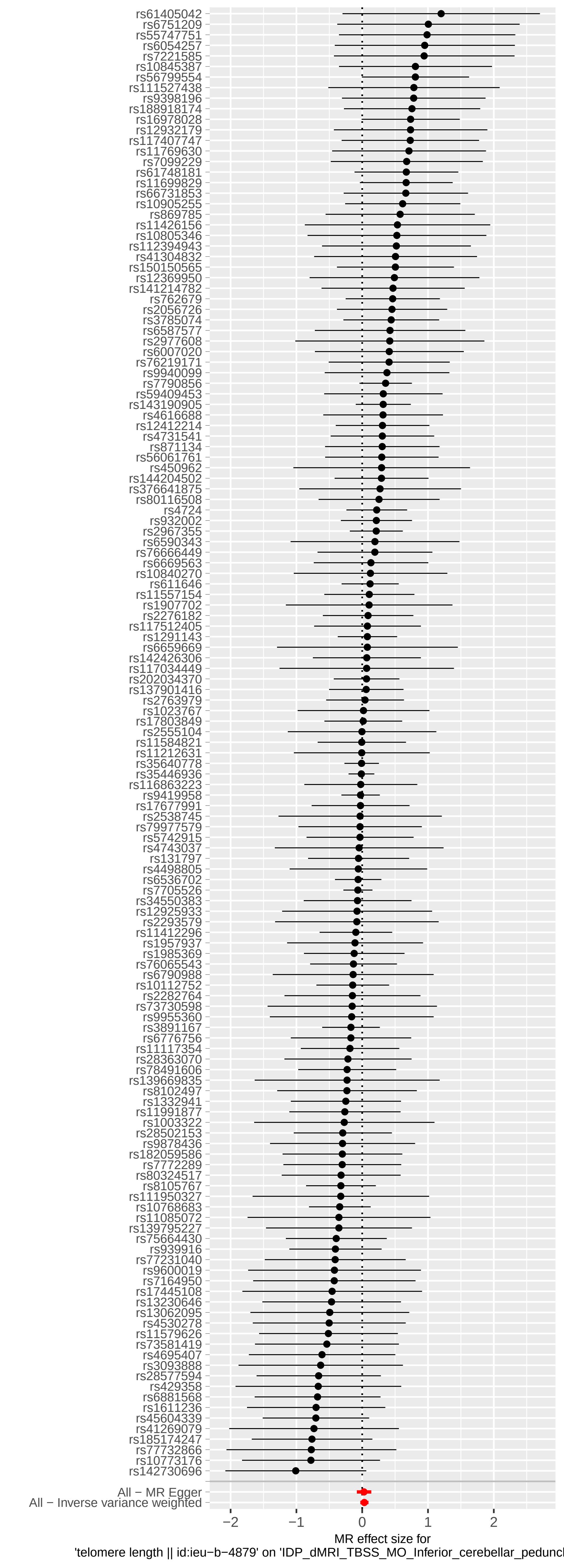
rs61405042
rs6751209
rs55747751
rs6054257
rs7221585
rs10845387
rs56799554
rs111527438
rs9398196
rs188918174
rs16978028
rs12932179
rs117407747
rs11769630
rs7099229
rs61748181
rs11699829
rs66731853
rs10905255
rs869785
rs11426156
rs10805346
rs112394943
rs41304832
rs150150565
rs12369950
rs141214782
rs762679
rs2056726
rs3785074
rs6587577
rs2977608
rs6007020
rs76219171
rs9940099
rs7790856
rs59409453
rs143190905
rs4616688
rs12412214
rs4731541
rs871134
rs56061761
rs450962
rs144204502
rs376641875
rs80116508
rs4724
rs932002
rs2967355
rs6590343
rs76666449
rs6669563
rs10840270
rs611646
rs11557154
rs1907702
rs2276182
rs117512405
rs1291143
rs6659669
rs142426306
rs117034449
rs202034370
rs137901416
rs2763979
rs1023767
rs17803849
rs2555104
rs11584821
rs11212631
rs35640778
rs35446936
rs116863223
rs9419958
rs17677991
rs2538745
rs79977579
rs5742915
rs4743037
rs131797
rs4498805
rs6536702
rs7705526
rs34550383
rs12925933
rs2293579
rs11412296
rs1957937
rs1985369
rs76065543
rs6790988
rs10112752
rs2282764
rs73730598
rs9955360
rs3891167
rs6776756
rs11117354
rs28363070
rs78491606
rs139669835
rs8102497
rs1332941
rs11991877
rs1003322
rs28502153
rs9878436
rs182059586
rs7772289
rs80324517
rs8105767
rs111950327
rs10768683
rs11085072
rs139795227
rs75664430
rs939916
rs77231040
rs9600019
rs7164950
rs17445108
rs13230646
rs13062095
rs4530278
rs11579626
rs73581419
rs4695407
rs3093888
rs28577594
rs429358
rs6881568
rs1611236
rs45604339
rs41269079
rs185174247
rs77732866
rs10773176
rs142730696
All − MR Egger
All − Inverse variance weighted
−2
−1
0
1
2
MR effect size for
'telomere length || id:ieu−b−4879' on 'IDP_dMRI_TBSS_MO_Inferior_cerebellar_peduncl
